# Proposed ANFIS Based Approach for Fault Tracking, Detection, Clearing and Rearrangement for Photovoltaic System

**DOI:** 10.3390/s21072269

**Published:** 2021-03-24

**Authors:** Ahmed F. Bendary, Almoataz Y. Abdelaziz, Mohamed M. Ismail, Karar Mahmoud, Matti Lehtonen, Mohamed M. F. Darwish

**Affiliations:** 1 Department of Electrical Power and Machines Engineering, Faculty of Engineering, Helwan University, Cairo 11795, Egypt; ahmed.bendary@h-eng.helwan.edu.eg (A.F.B.); ecc@ecc-consultant.com (M.M.I.); 2 Faculty of Engineering and Technology, Future University in Egypt, Cairo 11835, Egypt; almoataz.abdelaziz@fue.edu.eg; 3 Department of Electrical Engineering and Automation, School of Electrical Engineering, Aalto University, FI-00076 Espoo, Finland; karar.mostafa@aalto.fi (K.M.); matti.lehtonen@aalto.fi (M.L.); 4 Department of Electrical Engineering, Faculty of Engineering, Aswan University, Aswan 81542, Egypt; 5 Department of Electrical Engineering, Shoubra Faculty of Engineering, Benha University, Cairo 11629, Egypt

**Keywords:** PV arrays, fault detection, ANFIS, data analysis, module rearrangement

## Abstract

In the last few decades, photovoltaics have contributed deeply to electric power networks due to their economic and technical benefits. Typically, photovoltaic systems are widely used and implemented in many fields like electric vehicles, homes, and satellites. One of the biggest problems that face the relatability and stability of the electrical power system is the loss of one of the photovoltaic modules. In other words, fault detection methods designed for photovoltaic systems are required to not only diagnose but also clear such undesirable faults to improve the reliability and efficiency of solar farms. Accordingly, the loss of any module leads to a decrease in the efficiency of the overall system. To avoid this issue, this paper proposes an optimum solution for fault finding, tracking, and clearing in an effective manner. Specifically, this proposed approach is done by developing one of the most promising techniques of artificial intelligence called the adaptive neuro-fuzzy inference system. The proposed fault detection approach is based on associating the actual measured values of current and voltage with respect to the trained historical values for this parameter while considering the ambient changes in conditions including irradiation and temperature. Two adaptive neuro-fuzzy inference system-based controllers are proposed: (1) the first one is utilized to detect the faulted string and (2) the other one is utilized for detecting the exact faulted group in the photovoltaic array. The utilized model was installed using a configuration of 4 × 4 photovoltaic arrays that are connected through several switches, besides four ammeters and four voltmeters. This study is implemented using MATLAB/Simulink and the simulation results are presented to show the validity of the proposed technique. The simulation results demonstrate the innovation of this study while proving the effective and high performance of the proposed adaptive neuro-fuzzy inference system-based approach in fault tracking, detection, clearing, and rearrangement for practical photovoltaic systems.

## 1. Introduction

### 1.1. Motivation

One of the most promising fields in power plant generation is the generation of electrical energy using a renewable resource. Photovoltaic modules are considered the most widely used system in this field due to their advantages concerning low running cost and easy maintenance [1,2,3,4,5]. Some problems face the operation of these systems which lead to the high decrease of the whole system efficiency, one of the most common problems which attract the attention of many researchers is fault detection and clearing happening in one of these large number assembled modules [6,7,8,9,10]. Nevertheless, fault detection in several Photovoltaic (PV) systems remains a manually handled challenge in industrial applications, which requires a lot of attention. The overall global capacity of PV has noticed almost exponential progress in the earlier periods, increasing from 39 GWp in 2010 to 480 GWp in the year 2018, while the distinctive PV connection costs dropping from 4621 USD/kWp to 1210 USD/kWp for a similar duration [11]. For instance, European Union (EU) tracks an ambitious plan to be the world leader in the area of renewable energy sources by 2030 [12]. In Finland, the share of renewables is about 47% of all generation in the year 2018, counting wind energy, PV generation, and hydropower stations [13].

### 1.2. Literature Review

Many papers have been subjected to the fault tracking problem using different optimization control techniques [14]. Reference [15] presented the designing principle of a fault-tolerant PV system as the proposed technique succeeds in restricting the fault between some modules containing healthy and faulted PV cells. Further, [16] introduced various fault occurrences in a PV plant by explaining the limitations of existing detection and suppression techniques. The prescribed system was subjected to different fault proposed detection techniques and it was concluded that there is no universal fault detection technique that can detect and classify all faults in a PV system. Moreover, [17] obtained fault detection in PV modules through an algorithm formulated as a clustering problem using the robust minimum covariance determinant (MCD) estimator to describe its performance on simulated instances of arc and ground faults. Furthermore, [18] presents fault detection based on the comparison between the measured and model prediction results of the AC power production. In order to identify faults, a number of fault detection algorithms are based on the comparison between measured and modeled PV system outputs are implemented. In addition, [19] displayed how Arduino is used to monitor and detect faults in PV panels individually to avoid the simulation and modeling of the PV system, then the data is transmitted and interfaced through LabVIEW which compares the actual measured data and the reference data at the same condition. The authors of [20] showed a new approach for fault detection due to either detection of shading of PV modules and faults on the direct current (DC) side of PV systems.

Additionally, [21] presented an investigation of thermal monitoring of the PV solar modules and realize image processing by thermal radiation on PV modules in order to detect the broken cells on the PV panel and compare it with reference taken sample images on a panel within a periodic time interval. The authors of [22] demonstrated a fault detection procedure in order to completely avoid the use of modeling and simulation of the PV system through defining two new current and voltage indicators, NRc, and NRv, respectively, in the DC side of the inverter of the PV system, this method is based on the evaluation of these indicators. Additionally, [23] presented the applying of signal processing techniques to monitoring and control PV arrays. Fault detection algorithm objective function is developed as a combination problem and addressed using the robust minimum covariance determinant. Further, [24] showed many algorithms for fault detection algorithms are based on the comparison between measured output values and the reference modeled PV system outputs to determine the faults. It is established that metaheuristics, fuzzy logic, and artificial intelligence (AI) can provide improved performance in universal engineering applications [25,26,27,28,29,30,31,32]. Finally, References [33,34,35] described other approaches that use AI techniques, such as neural networks, fuzzy logic, and expert systems. However, all the above papers present many techniques and algorithms, but it suffers from many limitations like determining the existence of PV cell faults without locating these faults, additional equipment (e.g., signal generators, signal analysis devices) is needed to perform fault diagnosis [36]. 

### 1.3. Contributions

As stated above, faults in PV systems are nearly undetectable, especially under low irradiance conditions. If this fault remains hidden, it can significantly lesser the harvesting energy of solar arrays, damage the PV panels, and possibly reason fire threats. In this work, a new approach is developed for fault detection through a simple and low complexity fault detection algorithm as well as reconfiguration for the faulted PV panel to maintain the maximum achievable efficiency of the PV system. The novelty of this work is the introduction of two adaptive neuro-fuzzy inference system (ANFIS) based controllers where the first one is to detect the faulted string, while the other one is utilized for detecting the exact faulted group in the PV array. The fault detection approach is based on comparing the actual measured values of current and voltage and the trained historical values for this parameter, taking into consideration the ambient changed conditions like irradiation and temperature. The used model was installed using a configuration of 4X4 photovoltaic arrays connected through many switches in addition to four ammeters and four voltmeters. This case study is applied using MATLAB/Simulink where the simulations are presented to show the validity of the proposed approach. These simulation results prove the effectiveness and applicability of the proposed ANFIS based approach for not only fault tracking and detection, but also clearing, and rearrangement for real-world PV systems.

## 2. Problem Formulation and System Modelling

The main objective of this paper is to design a tracking system for fault algorithms in a PV array by implementing an artificial intelligence optimization technique to detect the exact location of the faulted PV cell. Each PV cell has its I-V and P-V output characteristics at the same temperature and different solar irradiance levels. The PV cell exhibits a non-linear output current and voltage relationship.

**Figure 1:**
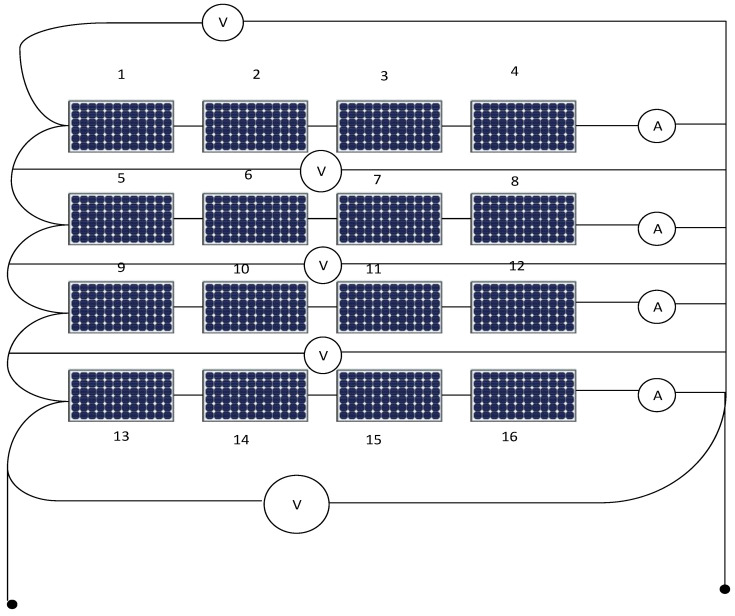
Photovoltaic array arrangement.

**Figure 2:**
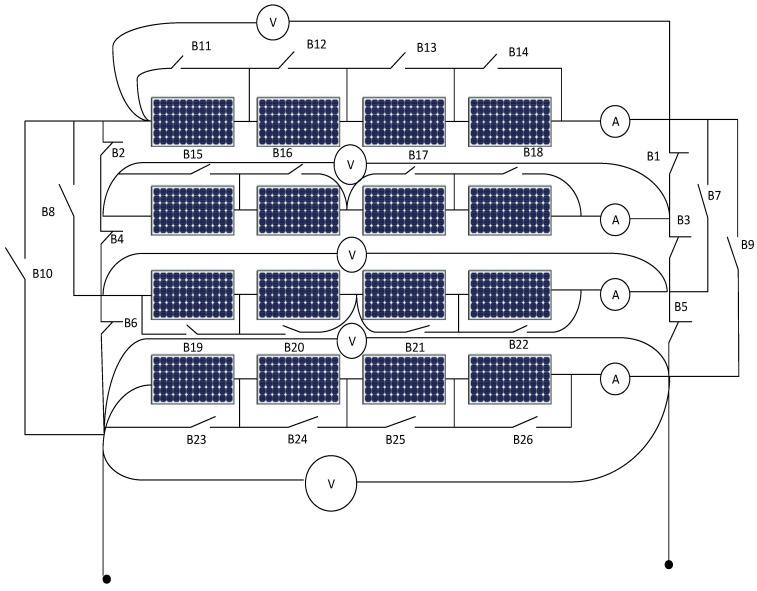
Connected switches for rearrangement of photovoltaic array system.

Figure 1 shows the system under study, it consists of 4 × 4 photovoltaic arrays where there are four PV cell groups connected in series, and each PV cell group has four PV cells connected in parallel, each string includes a series ammeter and parallel voltmeter. Further, in Figure 2, parallel switches among every panel in addition to extra switches like B1, B3, B5, B7, B8, B9, B10 are connected for enabling the rearrangement process using ANFIS controller action.

The rated current and voltage of each PV panel are known at a certain temperature and irradiation conditions and the reading of both ammeter and voltmeter is easily predicted through the above-described characteristic of each PV cell as well as the value of voltmeter and ammeter readings can be estimated after the outage of any PV cell and developing the clearing fault action through training these data using ANFIS.

## 3. Adaptive Neuro-Fuzzy Inference System (ANFIS)

**Figure 3:**
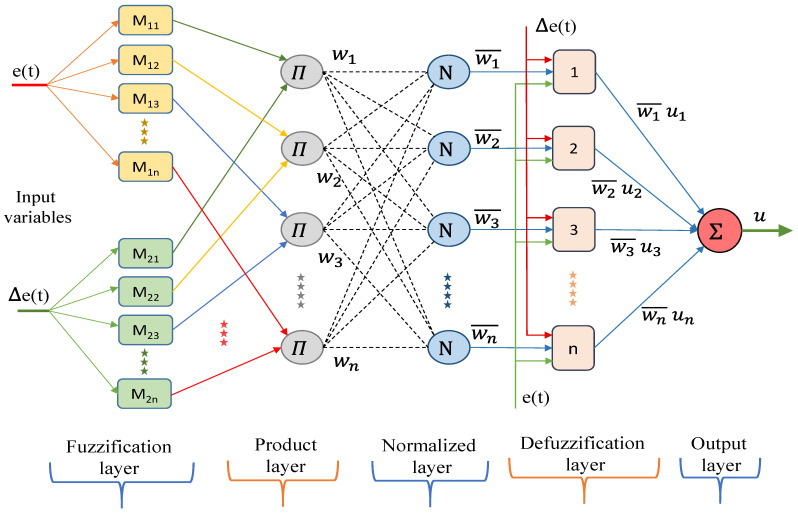
Schematic diagram of adaptive neuro-fuzzy inference system (ANFIS) architecture.

In this paper, the use of ANFIS is introduced. ANFIS is a cross between an Artifical Neural Network (ANN) and a fuzzy logic inference system. An artificial NN is intended to mimic the attributes of the human brain and comprises a gathering of artificial neurons. An adaptive system is a multi-layer feed-forward in which every node (neuron) plays out a capacity on input signals. More details about ANFIS structure can be obtained in [37,38,39]. Figure 3 shows the schematic diagram of the ANFIS structure. In this figure, the fixed nodes are represented by a circle while the adaptive node is represented by a square.

The ANFIS technique uses the Sugeno fuzzy model [40], where the fuzzy if–then rules are formulated by:

(1) $$R_{n}={\mathrm{if}\;M}_{1i}\left(e\right){\mathrm{and}\;M}_{2i}\left(\Delta e\right),\mathrm{then}\;f=p_{n}e\left(t\right)+q_{n}\;\Delta e\left(t\right)+r_{n}$$

where n represents the number of rules. Note that $M_{1i}$ and ${\;M}_{2i}$ represent fuzzy membership functions. $p_{n}$, $q_{n}$, and $r_{n}$ represent the linear parts of the corresponding n^th^ rule. 

Note that the first ANFIS layer involves the basic fuzzification where the degrees of membership functions are provided using the input variable. Typically, every node in the layer represents an adaptive function formulated by [38]:

(2) $$M_{1i}=\frac{1}{1+{\left[\frac{x-c_{i}}{a_{i}}\right]}^{b_{i}}}$$

where ($a_{i},{\;b}_{i},{\;c}_{i}$) represent the parameter set. Note that layer 2 stands for the product inference layer in which each node called with Π is under the firing strength of a particular fuzzy rule. Note that the outputs $w_{i}$ of the layer is represented as follows:

(3) $$w_{i}=M_{1i}\left(e\right)\times {\;M}_{2i}\left(\Delta e\right)$$

In turn, the third layer represents a normalization one, whereas the calculated firing strength from the preceding layer is normalized:

(4) $${\bar{w}}_{i}=\frac{w_{i}}{\sum_{i}(w_{i})}$$

Layer 4 receipts the normalized values from the third layer. Note that every node in this corresponding layer represents an adaptive mode (defuzzification) with a node function defined as [38]:

(5) $${\bar{w}}_{i}u={\bar{w}}_{i}\left(p_{i}e+q_{i}\Delta e+r_{i}\right)$$

in which (p, q, r) is the consequence parameter set while u represents the adopted control signal. Note that at the last layer, it is required to compute the summation of all inward signals to collect the output resulting portion of rules [40]:

(6) $$\underset{i\;}{\sum }({\bar{w}}_{i}u)=\frac{\sum_{i}w_{i}u}{\sum_{i}w_{i}}$$

## 4. Controller Design

Artificial intelligence (AI) control offers a way of dealing with modeling problems by implementing linguistic, non-formal control laws derived from expert knowledge. Many techniques have recently been used in the controller design of fault tracking systems, one of the most promising techniques is by using an ANFIS controller. In this paper, the network was trained to recognize the relationships between the input and output parameters, and the developed PV model is used to collect the training data. The operating temperature is varied from 15 °C to 45 °C in a step of 5 °C and the solar irradiance level is varied from 100 W/m^2^ to 1000 W/m^2^ in a step of 50 W/m^2^, to get the training data sets for ANFIS. 

**Figure 4:**
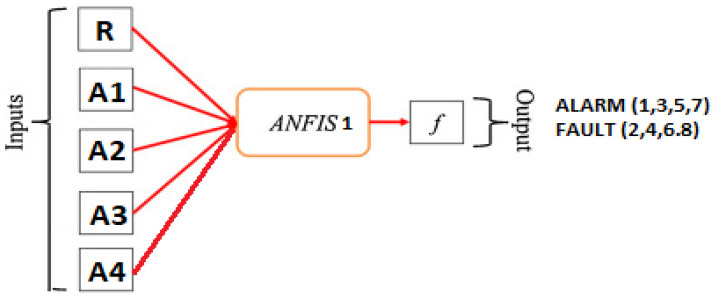
Block diagram of ANFIS controller 1.

In this paper two controllers based on ANFIS are developed, the first ANFIS controller shown in Figure 4 is working as either an alarm indicator for fault occurrence or for switching action purposes in case of a real existing fault. Such that, R is solar radiation, and A1, A2, A3, A4 are the meter readings for strings 1 to 4, respectively. The inputs for the first controller are irradiation, temperature, and the ammeter current values for each string of PV modules, also the output values of this controller are specified by values 1, 3, 5, and 7 for the alarm which gives an early indication for the predictive maintenance for the PV string 1, 2, 3 and 4, respectively, and the values 2, 4, 6 and 8 for the tripping action in case of string fault occurrence of PV string 1, 2, 3 and 4, respectively. For example, in the case of detecting a fault in PV string 1, the ANFIS controller output will be 2, and in this case, switches B1 and B2 will be disconnected to isolate the faulted string. This action will be repeated in case of a second, third, or fourth PV string fault. The main purpose of isolating the faulted string is to be preparing the string for the next test to identify exactly which group is faulted in the string.

**Figure 5:**
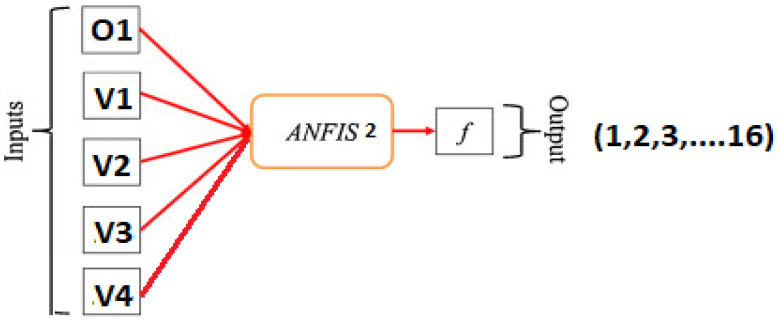
Block diagram of ANFIS Controller 2.

The main purpose of the second ANFIS controller shown in Figure 5 is to identify the exact location of the specified faulted panel in any PV string, then shorting it as a fault clearance action and for rearranging the PV array system to improve the efficiency of the PV system. 

The inputs of the second ANFIS controller are the output of the first ANFIS controller (2, 4, 6, 8) in addition to the readings of the voltmeter for each PV string. The second ANFIS controller will start its action after isolating the faulted PV string. The output of the second ANFIS controller is the location of the faulted panel (1, 2, 3, …, 16) based on panel location in the PV array as shown in Figure 1 and omitting it outside the system. Such that, O1 is the output of ANFIS 1, V1, V2, V3, and V4 are the voltmeter readings for string 1 to 4, respectively. Switches B1, B3, B7, and B9 are returned back the disconnected string again after isolating the faulted panel in the string to the system to improve the overall efficiency of the PV array system.

## 5. Simulation Results

### 5.1. Case Study

Simulation is done on a PV array system. The PV array consists of four PV cell groups connected in series, and each PV cell group has four PV cells connected in parallel. Each group consists of an equal number of series and parallel PV cells. There are constant series and parallel resistances for healthy PV groups (Rs and Rr respectively). There are an equivalent series and parallel resistances for the PV array system. The simulation inputs for the combined ANFIS controller 1 and 2 are R which represents solar radiation. Other inputs are A1, A2, A3, and A4 that denote the meter readings for PV strings 1 to 4, respectively. 

The variation in the resistance of PV cells is an indication of deterioration in its characteristics and may lead to fault occurrence in the future. This variation in cell resistance will lead to slight variation in the equivalent circuit of the PV array system. In case of a short circuit or open circuit occurring on a PV cell or group, the series and parallel equivalent circuit resistance of the PV array will be varied. The percentage value of resistance variation will lead to a variation in the characteristic curves of cells and arrays. The variation of the characteristic curves will be detected by the ANFIS controllers. The controllers will detect the damage groups that will cause an open circuit and short it, thereby giving the alarm as predictive maintenance in case of physical variation in the characteristics of PV cells. A test is done by varying the series and parallel resistance of a certain PV group of cells. Note that the input reference for the controller is the healthy model without fault which is used for ANFIS adaptation. 

**Figure 6:**
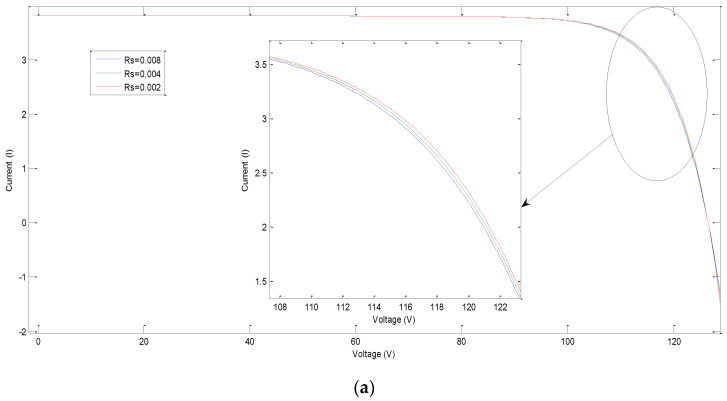

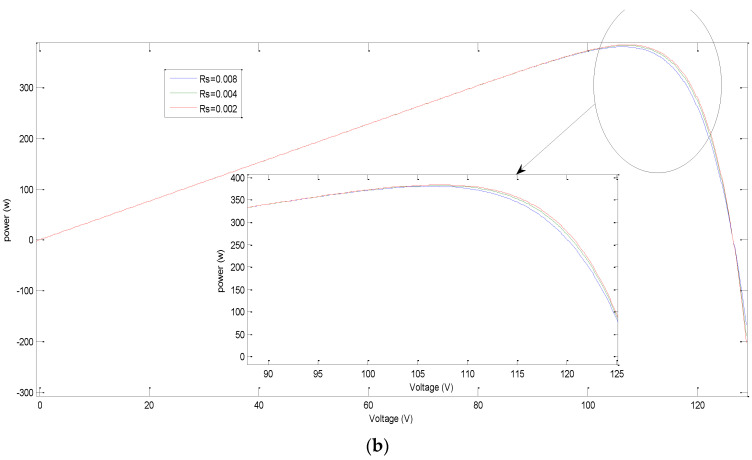
Effect of variation of series resistance on the characteristic of Photovoltaic (PV) cell: (**a**) I-V ch/s; and (**b**) P-V ch/s.

**Figure 7:**
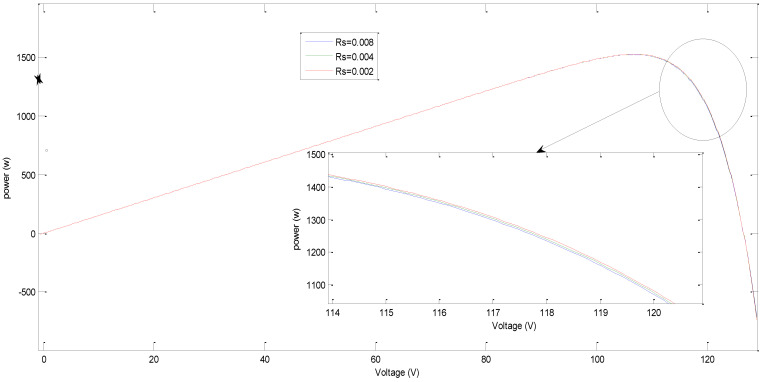
Effect of variation of PV cell series resistance on the power output of PV array.

**Figure 8:**
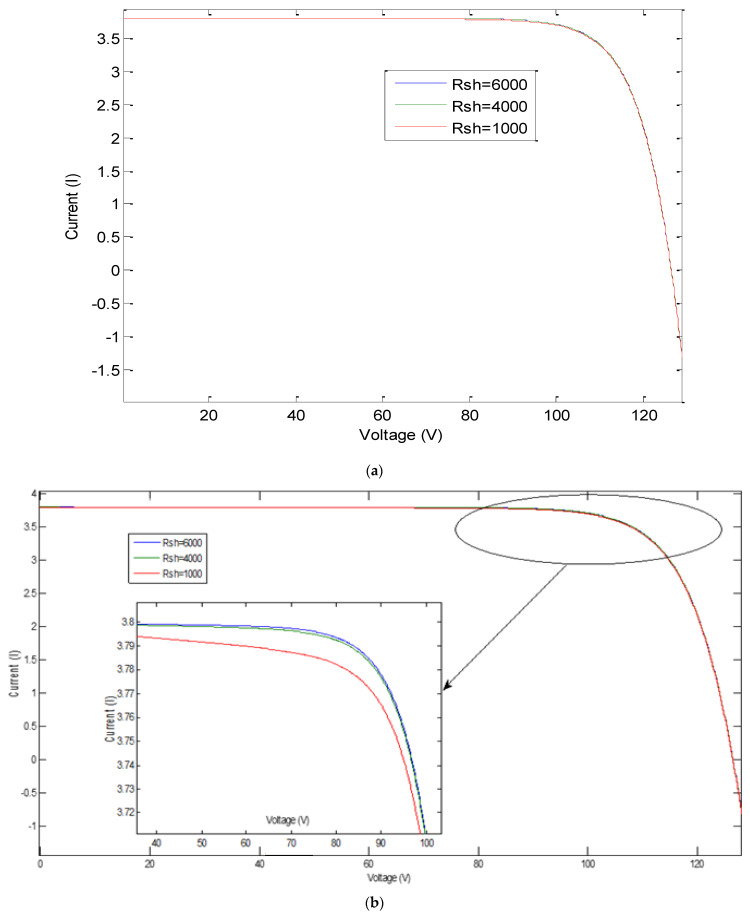

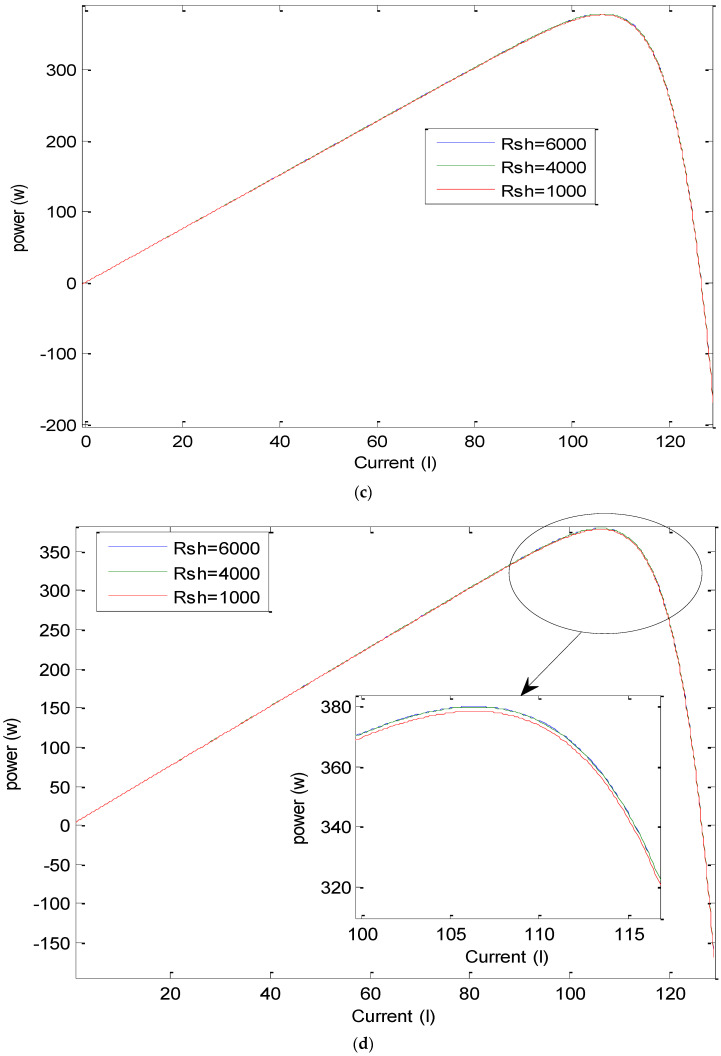
Effect of variation of shunt resistance on characteristic of PV cell: (**a**) I-V ch/s; (**b**) zoom in I-V ch/s; (**c**) P-I ch/s; and (**d**) zoom in P-I ch/s.

**Figure 9:**
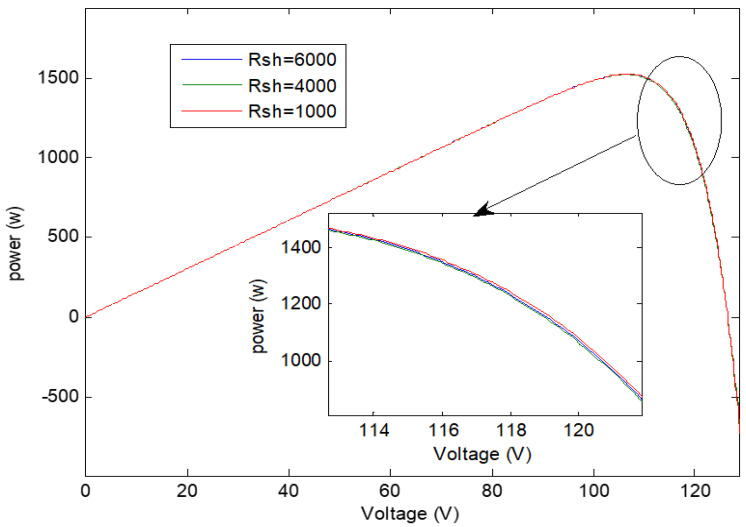
Effect of variation of PV cell parallel resistance on the power output of PV array.

The simulation result shows the effect of this variation on PV characteristics, groups, and arrays. The results are shown in Figure 6, Figure 7, Figure 8 and Figure 9, where R_sh_ is the equivalent shunt resistance of PV matrix modules. In this test, different Rs values (0.002, 0.004, and 0.008) are adopted in this simulation. Figure 6 shows the effect of variation of series resistance due to fault occurrence in the PV cell where the I-V and P-V characteristics are presented. Figure 7 demonstrates the impact of variation of series resistance due to fault occurrence in the proposed PV array system in which the P-V characteristic is presented. It is obvious that the harvesting PV power is the highest with a 0.002 Rs value compared to the other ones. Similarly, Figure 8a,b shows the effect of variation of shunt resistance (1000, 4000, and 6000) due to fault occurrence in the proposed PV cell, and the I-V characteristic is presented for different faults. In turn, Figure 8c,d displays the effect of variation of shunt resistance due to fault occurrence in the proposed PV cell, where the P-I characteristic is presented. Figure 9 shows the effect of variation of shunt resistance due to fault occurrence in the PV array, the P-V characteristic is presented for different faults.

**Figure 10:**
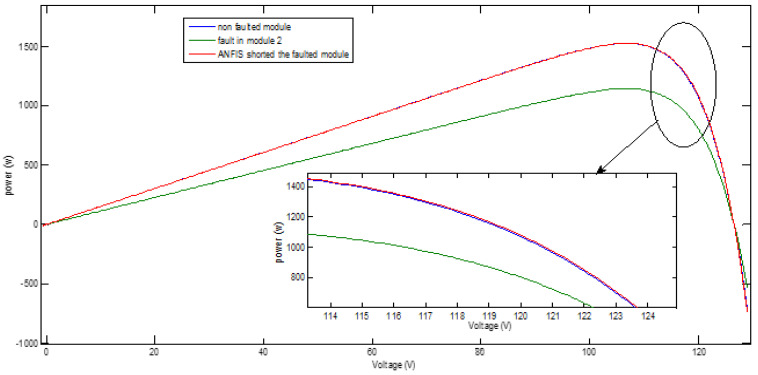
Effect of open-circuit fault occurred in a certain group on the power output of PV string.

**Figure 11:**
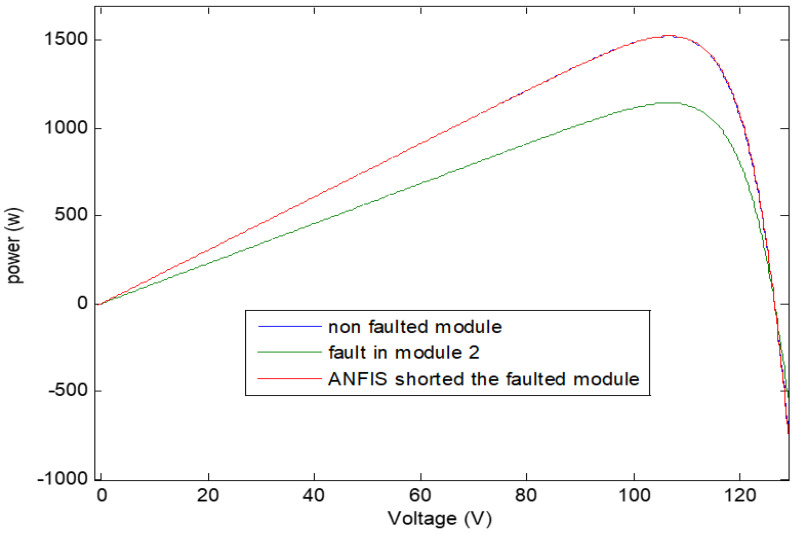
Effect of open-circuit fault occurred in a certain group on the power output of PV array.

**Figure 12:**
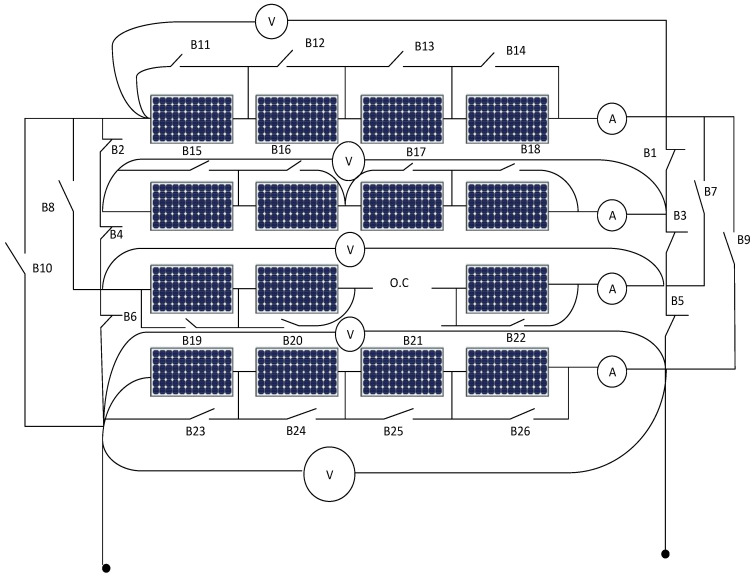
Construction of PV array during faulted PV group.

**Figure 13:**
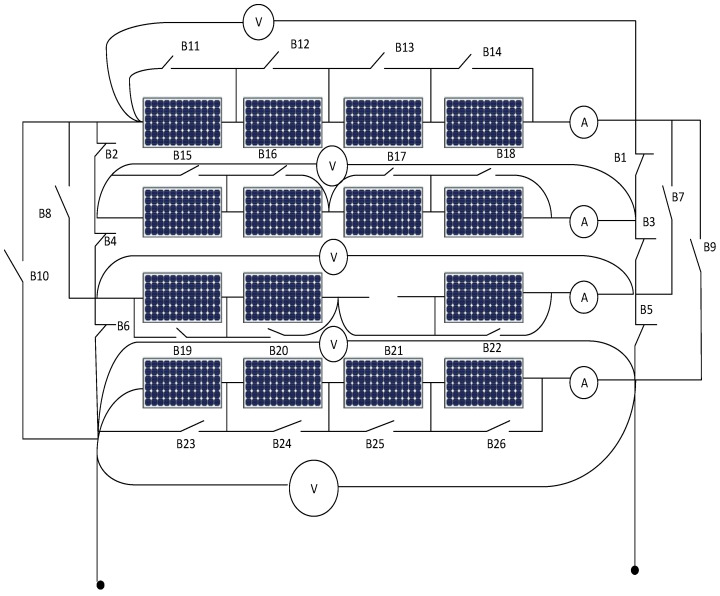
Reconstruction of PV array by shorting the faulted module using ANFIS controller.

A fault in group 21 occurred in the simulation model and becomes an open circuit. The ANFIS controller detected the fault and shorted the damaged module and the results of characteristic curve comparison are shown in Figure 10 and Figure 11 in cases of health, faulted conditions, and finally after shorting the faulted group. Specifically, the effects in P-V characteristic when the proposed system is exposed to open circuit fault and the ANFIS controller action for system restoration are shown in Figure 10 and Figure 11. The PV array structure during fault occurrence and after reconfiguration using the ANFIS controller is shown in Figure 12 and Figure 13. It is worth noting that the ANFIS controller can detect not only a single fault but also larger faults based on the input readings (radiation and current readings, and by referring to the readings of healthy PV matrix modules) which will require upgrading the training process of ANFIS.

### 5.2. Discussions

Table 1 gives a comparison between the efficiency of the array in the three cases for the same operating condition (same radiation and temperature) for 110 V DC load. As noticed in Table 1, the proposed method can attain the highest PV power that is 1490 W which is corresponding to 99.33% with respect to the normal case. In turn, the fault case without using the proposed rearrangement is only 1050 W which is corresponding to 70.00% with respect to the normal case. This analysis shows the significant enhancement of the harvesting energy from the PV systems under faulted conditions. This paper presents an optimum solution for fault finding and clearing, this is done through using artificially intelligent ANFIS. The new technique is established for fault detection by two ANFIS controllers used for this purpose. One of them is used to detect the faulted string and the other one is used for detecting the exact faulted group in the PV array.

Here, we compare the feature of the proposed work with the previous solutions. In [33], the authors have outlined an understanding of how advanced AI systems can operate by way of solving several problems in photovoltaic systems. Models of Sugeno fuzzy logic and PVSAT-2 have been developed to forecast the power production using solar irradiance in the array plane [34]. In [35], the ANFIS application for modeling and simulating PV power supply units has been introduced. Moreover, ANFIS has been implemented to model the delivered and consumed energy generation by PV, where numerous components of the global model have been accomplished using the dataset from the numerous input data of PV. In turn, in this work, we have introduced a new approach for fault detection by a simple and low complexity fault detection way, as well as reconfiguration for the faulted PV panel, thereby maintaining the highest achievable PV efficiency. In this paper, the comparison is done taking a base case as a fault without a controller, compared with the achievement approach when using the proposed controller as well as the model without fault. This work covers a gap in the literature, the novelty of this work is the proposal of two ANFIS based controllers. Specifically, the first ANFIS based controller is to detect the faulted string while the second ANFIS based controller is utilized for detecting the exact faulted group in PV. The controller can be easily designed and used in industry because the inputs are valid and easy to be measured (the inputs to the controller are the radiation, voltage, and current).

## 6. Conclusions

This paper presents an optimum solution for fault finding, tracking, and clearing. This is done by using one of the most promising techniques of artificial intelligence, e.g., ANFIS. Specifically, a novel approach is established for fault detection by a simple and low complexity fault detection method, as well as reconfiguration for the faulted PV panel for maintaining the maximum achievable efficiency of PV. In this regard, two ANFIS controllers are used for this purpose. One of them is used to detect the faulted string and the other one is used for detecting the exact faulted group in the PV array. The fault detection approach is based on the comparison between the actual measured values of current and voltage and the trained historical values for this parameter taking into consideration the ambient changed conditions including solar irradiation and temperature. This article considered the overcurrent (OC) fault in PV modules only that have a major effect on the performance of the PV array system. The results show a clear improvement in the efficiency of the PV array after the reconfiguration process. These simulation results verify the efficiency and applicability of the proposed ANFIS based technique for fault tracking, detection, as well as clearing, and rearrangement for PV arrays.

## Figures and Tables

**Table 1:** The efficiency of the PV array after rearrangement for the same operating condition.

PV Status	Power Output (W)
Normal	1500
Fault	1050
After rearrangement	1490

## Data Availability

The data presented in this study are available on request from the corresponding author.
